# Iliac Crest Bone Graft Harvesting: Modified Technique for Reduction of Complications

**DOI:** 10.31729/jnma.7086

**Published:** 2022-03-31

**Authors:** Rohit Kumar Pokharel, Sushil Paudel, Rajesh Bahadur Lakhey

**Affiliations:** 1 Department of Orthopaedics and Trauma Surgery, Institute of Medicine, Tribhuvan University Teaching Hospital, Maharajgunj, Kathmandu, Nepal

**Keywords:** *bone grafting*, *complications*, *iliac crest*, *pain*

## Abstract

Bone graft harvesting is one of the common procedures in orthopaedics surgery, and iliac crest is the gold standard donor site for autologous bone graft. There are a number of complications related with harvesting iliac crest bone graft, "donor site pain" is the commonest one. We modified the conventional surgical technique for autogenous iliac crest bone graft on patients who underwent anterior cervical decompression/ corpectomy and fusion surgeries. Among 23 patients, 18 didn't complain more pain at the donor site compared to the neck pain on the first postoperative day and the wound on the iliac crest did not affect their mobilisation. Mean Visual Analog Score was 2.62±1.80, 1.83±1.41, and 1.10±1.20 at the time of suture removal (14 days), at six weeks and three months respectively. At one year of follow-up, no patient complained of donor site pain. Our surgical modification has encouraging results and thus can be advocated for bone graft.

## INTRODUCTION

Autogenous grafts are safe and still standard globally as they have all three properties of bone graft, osteogenetic, osteoconductive and osteoinductive together with structural support.^1,2^ Iliac crest bone graft (ICBG) is the ideal source of autogenous bone graft.^3^ The tricortical graft as well as cancellous only grafts can be harvested from the iliac crest.^4,5^ Anterior ICBG is taken in anterior procedures like anterior cervical discectomy and fusion (ACDF) and anterior cervical corpectomy and fusion (ACCF); posterior ICBG is taken for posterior spinal fusion surgeries.^6–8^ We describe the improvement over the conventional technique of harvesting ICBG among patients undergoing ACDF/ACCF.

## MODIFICATION OF SURGICAL TECHNIQUE

**Figure 1:**
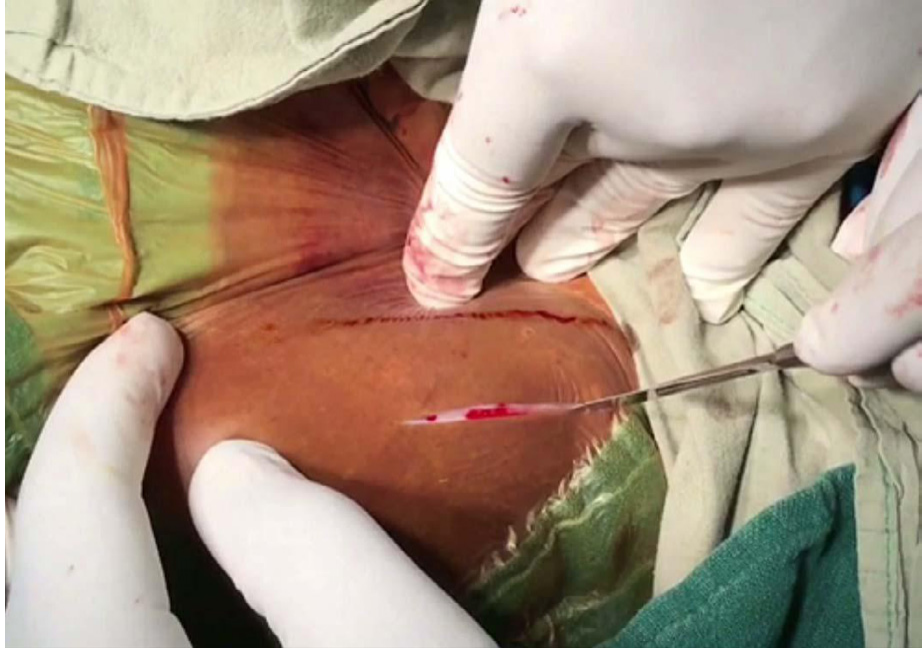
The intraoperative photograph shows the technique of stretching the skin towards the abdomen.

The patient was placed supine with a bolster over the required side gluteal region. Painting and draping is done as per the standard procedure. An incision is made, starting about three cm posterior to the anterior superior iliac spine, along the subcutaneous border of the iliac crest at the point of contact of the periosteum with attachments of the gluteal and trunk muscles after stretching the skin towards the abdomen (Figure 1).

The main aim of stretching the skin is to make sure that resultant scar does not form on the skin just overlying the bony prominent area. The incision length depends on the size of the graft needed and is deepened down to the lateral margin of the iliac crest. The soft tissue is cut along the margin of the crest; soft tissue attachment in the lateral surface of the iliac crest is stripped subperiosteally, keeping the attachments intact in the superior surface.

**Figure 2:**
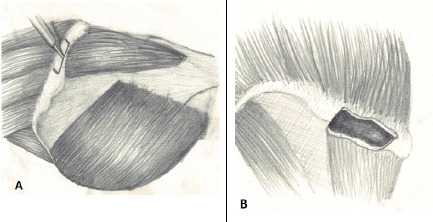
A) The incision is made on the outer margin of the iliac crest over the fascial attachment of muscles and periosteum, B) The iliac crest after elevating the periosteum together with a wafer of bone.

We harvest tri-cortical ICBG in cases of ACDF and ACCF for different clinical conditions. For tricortical grafts, depending on the size of the graft needed, soft tissue attachment is marked and cut transversely (lateral to medial) with a sharp osteotome up to one to two mm depth. Attachment of soft tissues in the upper surface of the iliac crest is lifted up together with a thin wafer of bone, then the iliacus muscle attachment in the medial surface of the iliac bone is cleaned down to a desired depth. The mayo-osseous flap (wafer of bone, periosteum, abdominal muscles and the iliacus muscle) is held with an Ellis forceps so that it does not get retracted (Figure 2). Desired size tricortical graft is harvested after cutting both tables of iliac bone on both sides with an osteotome or preferably, the power saw. Then with slight prying motions with a broad osteotome, the graft is peeled. Excessive heat is avoided by irrigating with saline at room temperature.

**Figure 3:**
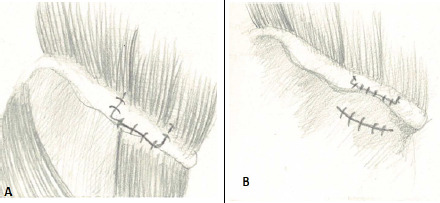
A) The periosteum with wafers of bone and muscles are opposed and sutured, B) After the suturing the incised skin. The external contour of the iliac crest is maintained and the skin suture is low lying.

When cancellous bone graft with one cortex is desired, thicker cortical bone of the superior cortex with its soft tissue attachment is raised without stripping the iliacus from the inner table. After lifting of the crest, considerable cancellous bone with the outer table of iliac bone is harvested in size as needed. If only cancellous bone is needed, the thicker cortical bone of the superior cortex with its soft tissue attachment is raised without stripping the iliacus from the inner table and gluteal muscles from the outer table of the iliac bone. Desired amount of cancellous bone is obtained by inserting a curette into the cancellous space. After harvesting the graft, the mayo-osseous flap is accurately opposed and sutured with interrupted sutures maintaining the contour of the iliac crest. The surgical skin wound is closed in layers and dressing is applied (Figure 3).

## OPERATIVE EVALUATION

We have evaluated 15 males and eight females who underwent surgery through the mentioned modified technique. The average age at the time of procedure was 35.70 years (range 21 to 72 years). Indications for surgery were: cervical prolapsed intervertebral disc (PIVD) with radiculopathy-eight patients, cervical spondylotic myelopathy-six patients (four single level and two level), unstable cervical fracture-five patients and cervical Pott's spine-four patients. Single level ACDF was performed in 15 cases (65.22%), two level ACDF was performed in two cases and ACCF was performed in six cases (four cases of infection and two cases of burst fractures).

Eighteen out of 23 (78.26%) cases did not complain more pain at the site compared to the neck pain on the first postoperative day, and the wound on the iliac crest did not affect their mobilisation. Mean Visual Analog Score (VAS) was 2.62 ±1.80 at the time of suture removal (14 days). It was 1.83±1.41 and 1.10±1.20 at six weeks and three months follow-up respectively. At one year of follow-up donor site pain was not complained (Table 1), and the patients could not readily appreciate the absence of the bone in the waist. The resultant skin scar falls away from the bony prominence resulting in less scar pain and superior cosmetic result. Two cases had hypertrophic scar at the incision site causing occasional itching. There were no cases of local nerve injury and hematoma formation. Two cases had superficial wound infection and were treated with regular dressing and antibiotics.

**Table 1:** Visual analog score at different follow-ups after the iliac crest bone graft for ACCF/ACDF.

Time of follow-up	Mean VAS score
14^th^ day (suture removal)	2.62±1.80
6 weeks	1.83±1.41
3 months	1.10±1.20
12 months	<1

## DISCUSSION

Iliac crest bone graft harvesting is associated with few known complications, frequency ranges from two to 49%. The complications associated ranges from simple bone pain to “landslide” hernia especially when full thickness grafts are taken.^9^ Commonest complication of ICBG is “donor site pain” and poor cosmetic appearance due to depression and scar in the iliac crest. Many patients complain of pain, which disturbed sleep within 1 month after surgery, and 13 to 20% of the patients experience chronic pain.^10–12^ Besides neural and vascular tissue injury, skin scar in the waist and 'donor site pain' are common complaints from the patient.^10–14^ There are some modifications in ICBG harvesting techniques to minimise these complications.^8,10,15^

Anterior iliac crest bone graft is usually harvested in anterior cervical spine surgery. Anterior Cervical \ Discectomy and Fusion (ACDF) and Anterior Cervical Corpectomy and Fusion (ACCF) are commonly performed operations in our hospital, indications being prolapsed cervical disc, single or double level cervical spondylotic myelopathy, infection, tumours, Ossification of Posterior Longitudinal Ligament (OPLL), deformity, and fracture of cervical spine. The gap created, is filled with bone (autograft or allograft).^16^ These days, there are many commercially available interbody spacers, made from a variety of materials, with their own advantages and disadvantages including availability, compatibility to radio-imaging and cost.^17–19^

Various techniques and implantation materials have been suggested to rebuild the iliac crest defect and to reduce the donor site pain. To avoid Iliac crest as donor site, Peelle MW, et al.^20^ chose manubrium sternae and Huang YC, et al.^21^ recommended proximal tibia as novel autologous source of bone graft. The reamer-irrigator-aspirator (RIA) is becoming popular device for harvesting cancellous only bone graft with significantly less donor site pain.^12,22^ Zhang J, et al. reconstructed iliac crest defect with bone cement and screws.^23^ Authors have primarily reconstructed the iliac crest with a hydroxyapatite-calcium triphosphate biphasic compound and bioactive ceramic spacers which improved the body's ability to reform new bone but did not alleviate the pain.^24,25^ Defino HL, et al. reconstructed ICBG donor site defect by using one of the patient's own ribs, which is feasible only in cases indicated for thoracotomy also.^26^ Gil-Albarova J, et al. used a transversal fence of thin tricortical chips obtained from the postero-lateral wall of the bone defect itself.^27^ Our technique of maintaining the iliac crest contour by keeping the mayo-fascial flap can also be used in posterior procedures in the spine.

## WAYS FORWARD

Modification in the surgical technique for iliac crest bone graft harvesting is an easier and cheaper procedure. The iliac crest contours were preserved with intact osseo-periosteo-muscle attachment without autologous bone or external materials. This technique showed less donor site pain with low-lying skin scar for better cosmesis.
